# P36 Diagnostic stewardship: improving antimicrobial susceptibility testing for *Campylobacter*

**DOI:** 10.1093/jacamr/dlaf230.043

**Published:** 2025-12-04

**Authors:** Jessica Smith, Lily Tian, Simon Dewar

**Affiliations:** NHS Lothian, Edinburgh, Scotland; NHS Lothian, Edinburgh, Scotland; NHS Lothian, Edinburgh, Scotland

## Abstract

**Background:**

Campylobacteriosis (largely attributed to enteric species *Campylobacter jejuni* and *Campylobacter coli*) is the most commonly reported gastrointestinal infection in Europe. It is usually a self-limiting enteritis, however, when treatment is required, macrolide antibiotics are the treatment of choice, due to low resistance rates and good clinical efficacy. Testing for *Campylobacter* and performing antimicrobial susceptibility testing (AST) can take up a reasonable amount of laboratory resources. This project aimed to improve this diagnostic pathway.

**Objectives:**

The objective was to identify the number of Campylobacter isolates in stool and blood cultures from NHS Lothian, particularly those that had AST. The analysis of this data aimed to evaluate laboratory practice and identify ways to optimize AST procedures.

**Methods:**

A retrospective analysis of Campylobacter isolates from enteric and blood cultures and the AST performed was conducted for the year 2023. Based on these findings, improvements were identified and laboratory practices, primarily relating to AST, were standardized and implemented in late 2024. A re-audit was then carried out from March to April 2025.

**Results:**

In 2023, a total of 1118 Campylobacter isolates were identified: 11 from blood cultures and 1107 from stool cultures. AST was performed on 70 of the 1107 (6.3%) enteric cultures and on all 11 (100%) blood cultures. All isolates tested were susceptible to erythromycin. The audit identified variations in both the methods used for AST (disc versus Etest) and the spectrum of antibiotics tested across Lothian, which highlighted the need for a standardized approach. Recommendations from the audit focused on two key areas: standardizing the AST methodology and reducing the number of AST performed for enteric cultures. A re-audit conducted in March and April 2025 identified 195 Campylobacter species in total. Of these, 193 were from stool cultures and two from blood culture samples. AST was performed on 3.1% (6/193) of enteric isolates, and the re-audit showed less variation in methodology. All isolates were again susceptible to erythromycin.

**Conclusions:**

This project successfully implemented diagnostic stewardship relevant to AST for Campylobacter laboratory samples. By standardizing AST methods and the spectrum of antibiotics tested, the number of AST performed on enteric samples was reduced by 50%. As the protocol becomes more embedded in laboratory practice, there is potential for further diagnostic stewardship outcomes.

